# Norovirus Polymerase Fidelity Contributes to Viral Transmission *In Vivo*

**DOI:** 10.1128/mSphere.00279-16

**Published:** 2016-10-19

**Authors:** Armando Arias, Lucy Thorne, Elsa Ghurburrun, Dalan Bailey, Ian Goodfellow

**Affiliations:** a Division of Virology, University of Cambridge, Addenbrooke’s Hospital, Cambridge, United Kingdom; b Institute of Immunology and Immunotherapy, University of Birmingham, Birmingham, United Kingdom; Boston University School of Medicine

**Keywords:** RNA polymerases, noroviruses, polymerase fidelity, quasispecies, virus transmission

## Abstract

Intrahost genetic diversity and replication error rates are intricately linked to RNA virus pathogenesis, with alterations in viral polymerase fidelity typically leading to attenuation during infections *in vivo*. We have previously shown that norovirus intrahost genetic diversity also influences viral pathogenesis using the murine norovirus model, as increasing viral mutation frequency using a mutagenic nucleoside resulted in clearance of a persistent infection in mice. Given the role of replication fidelity and genetic diversity in pathogenesis, we have now investigated whether polymerase fidelity can also impact virus transmission between susceptible hosts. We have identified a high-fidelity norovirus RNA-dependent RNA polymerase mutant (I391L) which displays delayed replication kinetics *in vivo* but not in cell culture. The I391L polymerase mutant also exhibited lower transmission rates between susceptible hosts than the wild-type virus and, most notably, another replication defective mutant that has wild-type levels of polymerase fidelity. These results provide the first experimental evidence that norovirus polymerase fidelity contributes to virus transmission between hosts and that maintaining diversity is important for the establishment of infection. This work supports the hypothesis that the reduced polymerase fidelity of the pandemic GII.4 human norovirus isolates may contribute to their global dominance.

**IMPORTANCE** Virus replication fidelity and hence the intrahost genetic diversity of viral populations are known to be intricately linked to viral pathogenesis and tropism as well as to immune and antiviral escape during infection. In this study, we investigated whether changes in replication fidelity can impact the ability of a virus to transmit between susceptible hosts by the use of a mouse model for norovirus. We show that a variant encoding a high-fidelity polymerase is transmitted less efficiently between mice than the wild-type strain. This constitutes the first experimental demonstration that the polymerase fidelity of viruses can impact transmission of infection in their natural hosts. These results provide further insight into potential reasons for the global emergence of pandemic human noroviruses that display alterations in the replication fidelity of their polymerases compared to nonpandemic strains.

## Abstract (precis)

Virus replication fidelity and hence the intrahost genetic diversity of viral populations are known to be intricately linked to viral pathogenesis and tropism as well as to immune and antiviral escape during infection. In this study, we investigated whether changes in replication fidelity can impact the ability of a virus to transmit between susceptible hosts by the use of a mouse model for norovirus. We show that a variant encoding a high-fidelity polymerase is transmitted less efficiently between mice than the wild-type strain. This constitutes the first experimental demonstration that the polymerase fidelity of viruses can impact transmission of infection in their natural hosts. These results provide further insight into potential reasons for the global emergence of pandemic human noroviruses that display alterations in the replication fidelity of their polymerases compared to nonpandemic strains.

## INTRODUCTION

Replication fidelity is a major trait influencing the pathogenesis of RNA viruses. The high error rates of viral RNA polymerases during genome replication result in genetically diverse virus populations, known as quasispecies, which are extremely flexible and adaptable to dynamic host environments and selection pressures (reviewed in reference 1). Alterations in replication fidelity typically lead to reduced virus infectivity in the host (2–5). This evidence has led to studies on the development of antiviral strategies based on the manipulation of virus replication fidelity. Such strategies have involved either the use of mutagenic compounds that can drive viral extinction through increased replication error rates (lethal mutagenesis; reviewed in references 6 and 7) or the design of mutant viruses with altered fidelity causing an attenuated phenotype *in vivo*, which can be used for the development of live vaccine candidates (4, 8, 9).

We recently demonstrated the antiviral activity of a novel mutagenic nucleoside, namely, T-705, or favipiravir, against persistent norovirus infections *in vivo* (10), providing the first link between norovirus polymerase fidelity and norovirus pathogenesis. Human noroviruses (HuNoVs) are considered the main cause of diarrheal episodes and foodborne gastroenteritis globally (11–13), with an estimated >500 M infections annually and >200,000 associated fatalities (13). Mortality rates are especially high in children under the age of 5 living in low-income countries, although other cohorts of vulnerable patients include elderly and immunocompromised populations (11, 14). HuNoV infections have also been linked to effects on an increasing number of other severe disorders such as exacerbation of inflammatory bowel disease, ulcerative colitis, and life-threatening chronic diarrhea (15, 16). Despite the significant impact on global health and the elevated economic losses associated with HuNoVs (11), there are no licensed vaccines or antiviral drugs available for the treatment of disease and control of outbreaks.

Since 2002, a sharp increase in the global incidence of norovirus outbreaks that is associated with the emergence of genogroup II.4 (GII.4) pandemic strains has been reported (17, 18). Evidence suggests that emerging HuNoV GII.4 pandemic strains transmit from person to person more efficiently than the previously dominant genogroup I strains (19, 20). Recent studies have demonstrated that the viral RNA-dependent RNA polymerases from pandemic HuNoV GII.4 strains display reduced replication fidelity and increased intrahost diversity in their resulting viral populations in comparison to nonpandemic strains (21). A newly emerging HuNoV GII.17 isolate, first identified in China, seems to be outcompeting the HuNoV GII.4 in some parts of Asia, resulting in increased outbreaks since the winter season of 2014 to 2015 (22). Recent studies suggest that this novel variant may be spreading globally, which could lead to the replacement of the pandemic genotype GII.4 by GII.17 (22–26). Fitting with the hypothesis that fidelity is linked to rapid emergence of norovirus isolates, the GII.17 isolate displays evolutionary rates at least 1 order of magnitude higher than those seen with GII.4 (25). Despite this evidence, a possible relationship between the reported reduced fidelity (and greater genetic diversity) in pandemic HuNoV GII.4 and the emerging GII.17 isolates and increased transmissibility has yet to be examined.

Here, we have assessed the influence of polymerase fidelity and genetic diversity on norovirus infectivity and transmission *in vivo* using a persistent strain of murine norovirus (MNV) as a model. MNV provides a robust surrogate system for studying HuNoV fidelity due to its high replication rates in cultured cells and to the availability of efficient reverse genetics systems and small-animal models (10, 27, 28). Here we have identified a high-fidelity MNV polymerase mutant (I391L) which shows delayed replication kinetics during the establishment of a persistent infection *in vivo*. Despite being able to successfully establish an infection and persist, the high-fidelity I391L mutant showed reduced mouse-to-mouse transmission compared to wild-type (WT) virus. Significantly, the transmission of a mutant with *in vivo* replication kinetics similar to those of the I391L mutant, but with WT-like polymerase fidelity (S313T), was unaffected, suggesting that the reduced transmission of I391L was linked to fidelity. These results support the role of genetic diversity as a significant trait influencing norovirus infectivity and transmission *in vivo* and provide new insight into the potential role of polymerase fidelity in the emergence of norovirus strains.

## RESULTS AND DISCUSSION

### Generation of MNV polymerase fidelity mutant candidates.

With the aim of identifying possible MNV polymerase (NS7) fidelity mutants, we carried out site-directed mutagenesis of residues previously identified as fidelity determinants in the orthologous positions of other viral RNA-dependent RNA polymerases. Given the substantial amount of data available on picornavirus polymerases and their relatively close genetic relationship with caliciviruses, we designed mutations in the MNV polymerase based on previous studies with poliovirus (PV), foot-and-mouth disease virus (FMDV), and coxsackievirus (CV) (3–5, 29–32). To date, the most paradigmatic example of viral attenuation as a result of increased fidelity is the PV polymerase (3D^pol^) G64S mutation, which resulted in a loss of pathogenicity with restricted virus tissue tropism in a lethal mouse model of PV infection (3, 4). While the norovirus polymerases do not share a great degree of structural homology with the PV 3D^pol^ in the domain surrounding the G64S position, MNV NS7 residue R77 (R74 in HuNoV NS7) lies within this domain and similarly establishes multiple hydrogen bond interactions with residues in the active site (30, 33, 34). The G64S change in PV 3D^pol^ is thought to alter fidelity by changing the hydrogen bond network, indirectly leading to subtle conformational changes of catalytic site residues (8, 30). Therefore, we examined the impact of substitutions at amino acid R77 on virus replication by reverse genetics (Table 1). All the mutations studied were lethal in the context of the MNV infectious clone, suggesting that this position is critical for polymerase function (Table 1). In order to identify tolerated MNV variants in this region of NS7, we mutated several neighboring amino acids of R77 that were not predicted to establish as many direct hydrogen bonds with amino acids involved in the active site and that would be predicted to result in more-subtle rearrangements of motif A. A series of positions, including V65, L70, P72, E75, and G78, were identified as possible tolerant sites within NS7 (Table 1). We also identified homologous positions in MNV NS7 based on other fidelity variants isolated in related picornaviruses, such as CV and FMDV, namely, K174, P175, S313, and I391 (Table 1). We successfully recovered mutants encoding NS7 substitutions P72S, E75S, K174R, K174S, S313T, I391L, and I391V, whereas the remaining mutants were nonviable (Table 1). The recovery of K174S and S313T yielded lower virus titers than the MNV WT, suggesting reduced replication fitness associated with these polymerase changes (Table 1). The stability of the viable mutants was confirmed by sequencing virus populations obtained after 4 serial passages in cell culture, with no additional changes identified within the NS7-coding region for any mutant (data not shown).

**TABLE 1:** Summary of the design and cell culture characterization of NS7 mutants

Mutant in orthologous viral polymerase^a^	Fidelity in orthologous virus polymerase^b^	MNV NS7 mutant^c^	MNV strain^d^	Viable in MNV^e^	Replication in cell culture^f^	Replication *in vivo*^g^
PV G64S	High	V65S	(1)	No		
		L70S	(1)	No		
		P72S	(1, 3)	Yes	Modest increase	Modest increase
		E75S	(1, 3)	Yes	Modest increase	WT
		R77A	(1)	No		
		R77K	(1)	No		
		R77N	(1)	No		
		R77S	(1)	No		
		G78S	(1)	No		
						
FMDV P169S/CV S164P	High/low	K174R	(3)	Yes	WT	WT
		K174S	(3)	Yes	Significantly decreased	Significantly decreased
		P175S	(3)	No		
						
CV S298T	Low	S313T	(3)	Yes	Modest decrease	Significantly decreased
						
CV A372V	Low	I391D	(3)	No		
		I391L	(3)	Yes	WT	Significantly decreased
		I391R	(3)	No		
		I391V	(3)	Yes	WT	WT
						
Isolated by passage in ribavirin		T35I	(3)	Yes	WT	Modest decrease (viral RNA)
						
Isolated by passage in ribavirin		V330I	(3)	Yes	WT	WT
						
Designed based on MNV V330I		V330A	(3)	No		
		V330S	(3)	No		

a Substitutions generated in this study were based on fidelity changes found in other viruses (4, 5, 29, 31, 32): G64S in PV; P169S in FMDV; and S164P, S298T, and A372V in CV.

b Effect on virus polymerase fidelity caused by substitutions shown in the first column.

c MNV NS7 polymerase mutants generated based on the fidelity of the residues shown in the first column. Mutants V330I and T35I were constructed after isolation of these mutations in MNV populations treated with increasing concentrations of ribavirin (see Fig. S1 in the supplemental material). Other variants of position 330 (V330A or -S) were also prepared based on the isolation of V330I during ribavirin treatment. These mutants were not viable.

d MNV strain. NS7 mutations were introduced in plasmids containing full MNV-1 or MNV-3 genome sequences. Hence, “(1)” denotes MNV-1, “(3)” denotes MNV-3, and “(1, 3)” denotes those mutations tested in both strains. For the experiments represented in the figures, only MNV-3 variants were used.

e The viability of NS7 mutants was assessed by reverse genetics after recovery in BHK-21 (or BHK-21-derived BSR-T7) cells followed by 3 serial passages in RAW264.7 cells as described in reference 27.

f Phenotype in cell culture. “Modest increase or decrease” (in virus replication) indicates statistically significant changes in virus titers of ≤1 log_10_ (two-way analysis of variance [ANOVA] test) at any given time point during replication kinetics. “Significantly decreased” in virus replication (K174S) refers to statistically significant changes in virus titers of ~2 log_10_ at an early replication time point. In this column, “P72S” and “E75S” refer to the corresponding NS7 mutants in the MNV-3 genome context.

g Phenotype *in vivo*. “Modest increase” (in virus replication) *in vivo* indicates a highly significant change in the titer of virus shed in feces (>1 log_10_; *P* < 0.001; two-way ANOVA). “Significantly decreased” indicates highly significant changes in levels of both viral RNA and infectious virus shed in feces (*P* < 0.001; ANOVA test). “Modest decrease (viral RNA)” indicates significantly lower RNA levels but not virus titers shed in mouse feces. In this column, “P72S” and “E75S” refer to the corresponding NS7 mutants in the MNV-3 genome context.

Since the propagation of different RNA viruses in cell culture in the presence of ribavirin generally leads to the selection of fidelity mutants (e.g., PV G64S, FMDV P169S, and CV A372V, among others) (29, 31, 32), we similarly investigated whether the passage of MNV in the presence of ribavirin also selected for changes in NS7 conferring higher polymerase fidelity. We identified substitutions T35I and V330I in NS7, which emerged in two lineages of MNV that had been repeatedly passaged in the presence of ribavirin (see Fig. S1 in the supplemental material). No substitutions were found in the NS7 RNA polymerase of MNV passaged in parallel in the absence of the drug. These mutations were introduced in the MNV infectious cDNA clone, and viable mutants were recovered (Table 1).

10.1128/mSphere.00279-16.1 Figure S1  Serial passage of MNV-3 in the presence of increasing concentrations of ribavirin. Data represent results of MNV-3 serial passages in the presence (solid lines containing symbols) or absence (dashed line, no symbols) of ribavirin. Each value is the average of results of three titer determinations, with the error bars representing SEM. Increasing concentrations of ribavirin (RBV) were used as indicated by different symbol colors: 200 µM, white; 400 µM, gray; 800 µM, black. The amino acid substitutions identified in the NS7-coding region for each lineage are provided on the right side of the graph. Download Figure S1, PDF file, 0.03 MB. Copyright © 2016 Arias et al. 2016 Arias et al. This content is distributed under the terms of the Creative Commons Attribution 4.0 International license.

### Substitution I391L results in increased polymerase fidelity.

**FIG 1:**
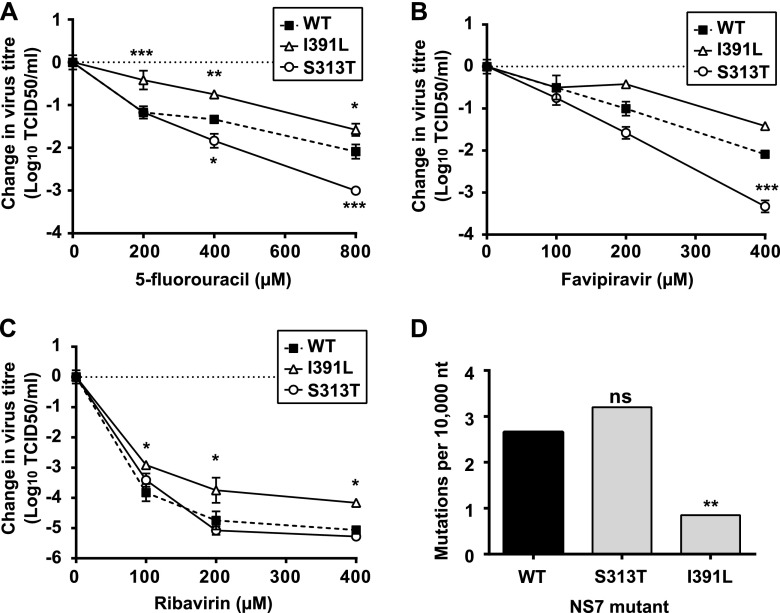
Substitution I391L in the MNV NS7 RNA polymerase leads to restricted genetic diversity in viral populations. (A to C) Sensitivity of the WT strain and mutants I391L and S313T to different mutagenic compounds: 5-fluorouracil (A), favipiravir (B), and ribavirin (C). Sensitivity to each mutagen is expressed as the change in virus titer relative to the value for infections in the absence of mutagens. Each value is the average of results of three independent biological replicates (*n* = 3), with the error bars representing standard errors of the means (SEM). An apparent absence of SEM bars indicates that the bar is smaller than the respective symbol for these values. Sensitivity values for each MNV NS7 mutant were compared with WT values. Hence, those mutants with titer change curves above the WT curve were less sensitive to drug treatment, whereas those with curves below the WT curve showed greater sensitivity. Statistical analysis of relative levels of sensitivity in each NS7 mutant compared to the WT was performed by two-way analysis of variance (ANOVA) (*, *P* < 0.05; **, *P* < 0.01; ***, *P* < 0.001). (D) Mutation frequency analysis of MNV populations obtained after 8 serial passages in RAW264.7 cells in the absence of mutagenic treatment. Viral RNA was extracted and amplified by RT followed by PCR as explained in Materials and Methods. Amplified cDNA molecules were ligated to PCR Blunt vector, and positive colonies containing MNV insertions were sequenced. Statistical significance in the genetic diversity of different mutant viruses (S313T and I391L) with respect to that of the WT was determined by a Mann-Whitney test, which was used to compare the ranked scores of the numbers of mutations found in individual clones grouped by population, as further explained in Materials and Methods (ns, not significant; **, *P* < 0.05). Repeated mutations were taken into consideration only once for the analysis, as previously described (10). These mutation frequencies correspond to the identification of 34, 23, and 10 unique mutations during the sequence analysis of 127,870, 71,844, and 117,540 nucleotides (nt) in molecular clones from WT, S313T, and I391L populations, respectively. The total numbers of clones analyzed (*n*) in this study were 60, 34, and 56 for the WT, S313T, and I391L populations, respectively.

As an indirect approach to identify any fidelity alterations in the viable MNV mutants, we tested the sensitivity of the viable mutants to the following three different nucleoside analogues with mutagenic activity against a variety of RNA viruses, including MNV: favipiravir, 5-fluoruracil, and ribavirin (3, 5, 10, 35). High-fidelity mutants are typically less sensitive to mutagenic treatment, while mutator (lower fidelity) polymerase variants are typically more sensitive (5, 29, 32). The I391L mutant had reduced sensitivity to all three mutagenic compounds, while the S313T mutant was more sensitive to 5-fluorouracil and favipiravir (Fig. 1). I391V sensitivity varied depending on the mutagenic compound, and the I391V mutant was more sensitive to 5-fluorouracil and favipiravir and less sensitive to ribavirin (see Fig. S2 in the supplemental material). The remaining mutant viruses showed no significant differences in their sensitivities to any of these mutagenic compounds compared to the WT (see Fig. S3).

10.1128/mSphere.00279-16.2 Figure S2  Substitution I391V in NS7 leads to altered levels of sensitivity to mutagens in MNV that are not accompanied by changes in genetic diversity. (A to C) Sensitivity of the WT and I391V strains to the following different mutagenic compounds: 5-fluorouracil (A), favipiravir (B), and ribavirin (C). The sensitivity to every mutagen is expressed as the change in virus titer relative to the value determined for infections in the absence of mutagens. Every value in the graph is the average of results of three independent infections (*n* = 3), with the error bars representing SEM. The absence of SEM bars in certain values indicates that the bar is smaller than the respective symbol. Sensitivity values for I391V mutant were compared with WT values. I391V curve plots above the WT curve indicate less sensitivity to drug (ribavirin), whereas a curve below the WT curve indicates greater sensitivity (favipiravir and 5-fluorouracil). Data represent results of a two-way analysis of variance (ANOVA) test (*, *P* < 0.05; **, *P* < 0.01; ***, *P* < 0.001). The dashed line in panels A to C represents the absence of change in relative virus titers. (D) Mutation frequency analysis of MNV populations obtained after 8 serial passages in RAW264.7 cells in the absence of mutagenic treatment. Viral RNA was extracted and amplified by RT followed by PCR; cDNA was ligated to PCR blunt vector, and positive colonies containing MNV insertions were sequenced as described in Materials and Methods. Statistical significance in the genetic diversity of I391V with respect to that of the WT was determined by the Mann-Whitney test as further explained in Materials and Methods (ns, not significant) and as previously described (10). Repeated mutations are taken into consideration only once for the analysis. These mutation frequencies correspond to the identification of 34 and 20 unique mutations during the sequence analysis of 127,870 and 72,242 nucleotides, respectively, for the WT and I391V populations. The total numbers of clones analyzed (*n*) in this study were 60 and 34 for the WT and I391L populations, respectively. Download Figure S2, PDF file, 0.04 MB. Copyright © 2016 Arias et al. 2016 Arias et al. This content is distributed under the terms of the Creative Commons Attribution 4.0 International license.

10.1128/mSphere.00279-16.3 Figure S3  Effect of NS7 mutations on virus sensitivity to different mutagenic compounds. (A) Sensitivity to 5-fluorouracil (FU) and ribavirin (RBV) in different recombinant MNV strains containing the described mutations in the MNV NS7 RNA-dependent RNA polymerase; the sensitivity of each mutant was measured as the change in virus titer between infections performed in the presence and absence of drugs. For clarity, these results have been expressed relative to WT results. A positive value (above the dashed line) indicates reduced sensitivity and a negative value (below the dashed line) greater sensitivity to each corresponding drug. (B) Sensitivity of MNV NS7 mutants to different concentrations of favipiravir (expressed as the change in virus titer observed between infections in the presence and absence of drug treatment). In both panels, every value represent the average of results of three independent biological replicates (*n* = 3). Error bars represent SEM, with the statistical significance determined by two-way ANOVA test (**, *P* < 0.01; ***, *P* < 0.001). Download Figure S3, PDF file, 0.05 MB. Copyright © 2016 Arias et al. 2016 Arias et al. This content is distributed under the terms of the Creative Commons Attribution 4.0 International license.

Given this pattern of sensitivity to different mutagenic nucleosides, substitutions I391L, I391V, and S313T were further examined for any impact on replication fidelity. Several studies have demonstrated that alterations in polymerase fidelity typically lead to changes in the genetic diversity of the resulting virus population (4, 5). With this in mind, we measured the genetic diversity in MNV NS7 mutant populations after their serial passage in cell culture in the absence of any mutagenic treatment. We found that the genetic diversity in the I391L NS7 mutant virus population was ~3-fold lower than in the WT NS7 population, suggesting that the I391L substitution results in a higher-fidelity NS7 (Fig. 1D; see also Table S1 in the supplemental material). The NS7 mutants S313T and I391V did not show any significant difference in their mutation frequencies compared to the WT, suggesting that their increased sensitivity to mutagens was not related to any impact on polymerase fidelity (Fig. 1D; see also Fig. S2D and Table S1). Hence, the S313T and I391V mutants display WT-like fidelity phenotypes.

10.1128/mSphere.00279-16.8 Table S1  Mutation frequency analysis of NS7 mutant virus populations. Download Table S1, DOCX file, 0.02 MB. Copyright © 2016 Arias et al. 2016 Arias et al. This content is distributed under the terms of the Creative Commons Attribution 4.0 International license.

### The high-fidelity I391L NS7 mutant shows delayed replication *in vivo* but not in cell culture.

**FIG 2:**
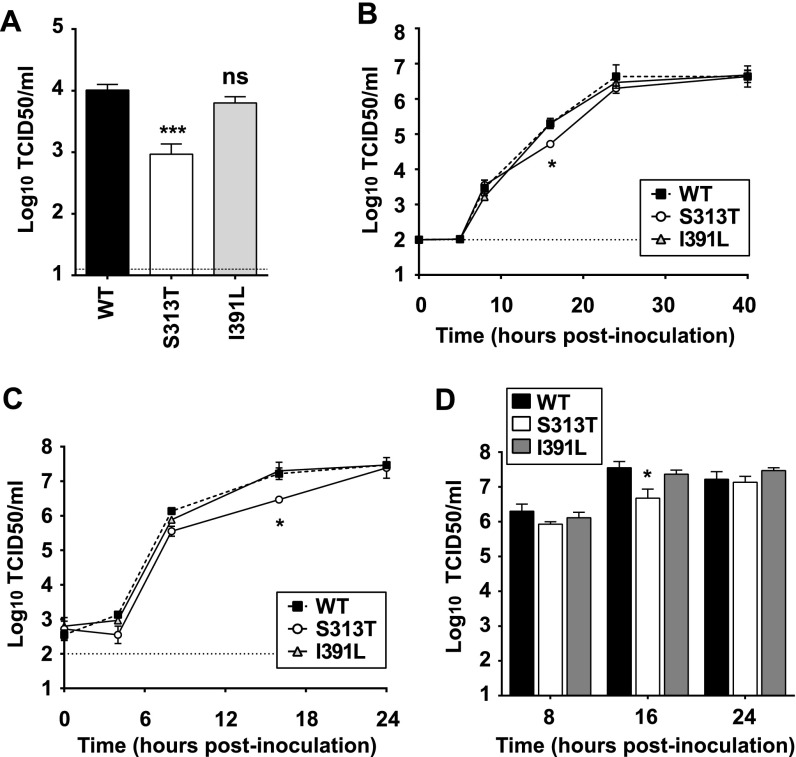
The MNV NS7 RNA polymerase mutant I391L shows WT-like replication kinetics during infection in cell culture. (A) Virus yield after reverse genetics recovery of full-length cDNA constructs containing either wild-type (WT) MNV or the polymerase mutants S313T and I391L. The values shown are the averages of results of at least three independent virus titer determinations for up to 4 independent cDNA transfections. Error bars represent SEM, with the statistical significance determined by one-way ANOVA (ns, not significant; ***, *P* < 0.001). (B to D) Replication kinetics of WT and mutant MNVs in RAW264.7 cells infected at the following different MOI’s: 0.01 TCID_50_/cell (B), 5 TCID_50_/cell (C), and 10 TCID_50_/cell (D). The values represent the averages of results of three independent biological replicates (*n* = 3), with the error bars representing SEM. Data were analyzed by two-way ANOVA (*, *P* < 0.05). The dashed line in panels A to C represents the limit of detection.

**FIG 3:**
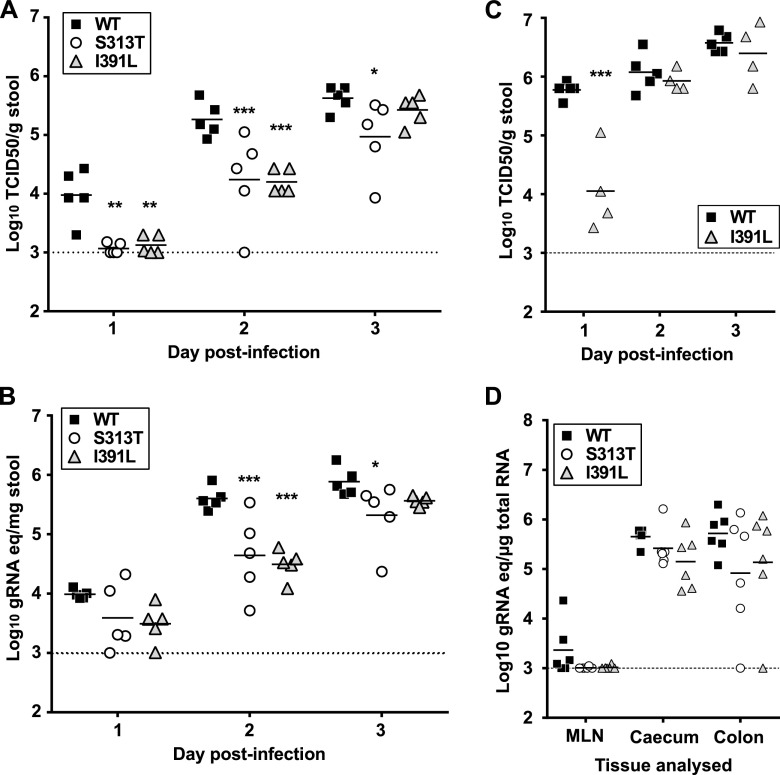
MNV NS7 RNA polymerase mutant I391L shows delayed replication kinetics in mice. (A and B) Virus titers (A) and viral RNA levels (B) shed in the feces of animals inoculated with 3 × 10^2^ TCID_50_ MNV (*n* = 5). Mice were inoculated with recombinant viruses generated following recovery of MNV NS7 variants in BHK-21 cells by reverse genetics followed by 4 serial infections in RAW264.7 cells, as previously described (39). The presence of the desired mutations in the viral genome was confirmed by sequencing. Data were analyzed by two-way ANOVA (*, *P* < 0.05; **, *P* < 0.01; ***, *P* < 0.001). gRNA eq, genomic RNA equivalents. (C) Animals were infected as described for panel A, but the mice were inoculated with a dose of 3 × 10^3^ TCID_50_ MNV per mouse (*n* = 5). Data represent results from a two-way ANOVA test (***, *P* < 0.001). (D) Viral RNA levels detected in various tissues collected from mice inoculated with 3 × 10^2^ TCID_50_ of WT MNV or the NS7 polymerase mutants at 48 h postinfection. MLN, mesenteric lymph node (two-way ANOVA test results were nonsignificant). In all panels, the dashed line represents the detection limit. The apparent absence of SEM bars for some values in panels B and C indicates that the bar is smaller than the respective symbol.

We investigated whether the increase in fidelity conferred by I391L resulted in any phenotypic alteration of norovirus replication *in vivo*, as previously documented for other viruses (4, 9). Prior to this, a more detailed characterization of the replication kinetics of the I391L mutant and the remaining NS7 mutants was performed in cell culture. The I391L mutant showed no significant difference from WT MNV in terms of the virus yields obtained following viral recovery by reverse genetics from a full-length cDNA construct (Fig. 2A). Similarly, no replication defect was observed at either a low multiplicity of infection (MOI) (0.01 50% tissue culture infective dose [TCID_50_]/cell; Fig. 2B) or a high MOI (5 to 10 TCID_50_/cell; Fig. 2C and D) for immortalized macrophage cells. However, I391L showed significantly delayed kinetics of replication *in vivo* as reflected in decreased viral RNA levels and lower titers of infectious virus secreted in the feces of the mice (Fig. 3A and B). This observation suggests that the higher replication fidelity of I391L results in reduced fitness during the establishment of a persistent infection *in vivo*. The early defect in I391L replication was recovered by day 3 postinfection (Fig. 3A and B) and then remained comparable to the WT level for the duration of the experiment (28 days; data not shown). Animals infected with a 10-fold-higher viral titer also showed delayed replication kinetics for I391L, although viral loads similar to the WT levels were again observed by day 2 (Fig. 3C). The observed lower virus titers and RNA levels in feces of mice infected with I391L were associated with a trend toward lower viral RNA levels in different tissues harvested at 48 h postinoculation, although the differences observed did not reach statistical significance (Fig. 3D).Taking the data together, the reduced replication kinetics of the MNV I391L NS7 mutant were apparent only *in vivo* and not during replication in cell culture. This is in line with observations of other reported fidelity mutants (4, 32).

Of the remaining NS7 mutants, S313T and K174S also displayed decreases in replication *in vivo* to levels similar to those seen with I391L (Fig. 3A and B; see also Fig. S4 in the supplemental material). However, in contrast to I391L, both S313T and K174S also displayed small but significant replication defects in cell culture. Both the S313T and K174S mutants yielded reduced virus titers in virus recoveries by reverse genetics (Fig. 2A; see also Fig. S4A) that were typical of viruses with a replication defect. K174S also showed significantly delayed kinetics during infection in RAW264.7 cells (see Fig. S4B). In contrast, the S313T mutant displayed minimally but reproducibly lower virus titers at 16 h postinfection during kinetics assays performed with both high and low MOI’s (Fig. 2B to D). The mutants I391V and T35I exhibited modest replication defects *in vivo*, although to a lesser extent than I391L (see Fig. S4 and S5). Surprisingly, significantly higher numbers of genome copies were secreted in mice infected with the P72S mutant, with slightly increased replication also being observed in cell cultures (see Fig. S6), suggesting that this substitution confers a replication advantage to MNV. Given that P72S did not differ in its sensitivities to mutagenic nucleosides, we hypothesized that the observed phenotype was not due to any impact on fidelity and it was therefore not characterized in more detail.

10.1128/mSphere.00279-16.4 Figure S4  *In* *vivo* and in-cell-culture replication of MNV NS7 mutants T35I, K174R, and K174S. (A) Viral titers obtained after reverse genetics recovery of MNV-3 WT and NS7 mutants following transfection of full-length cDNA constructs. The values are the average of the results of at least three independent virus titer determinations for up to 4 independent cDNA transfections (one-way ANOVA test). (B) Replication kinetics of the MNV-3 WT strain and mutants in RAW264.7 cells infected at an MOI of 5 TCID_50_/cell. Every value is the average of results of three independent biological replicates (*n* = 3). Error bars represent SEM, with the statistical significance determined by two-way ANOVA test. (C and D) Viral titers (C) and viral RNA levels (D) in the feces of mice infected with 3 × 10^3^ TCID_50_ units of various MNV WT or mutant strains at 24 h postinfection. Every value represent the average of the results from five samples obtained from independent animals (*n* = 5), with the statistical significance determined by a two-way ANOVA test. Statistical significance is represented as follows: *, *P* < 0.05; **, *P* < 0.01; ***, *P* < 0.001. In all the panels, the dashed line indicates the limit of detection, and the error bars represent SEM. The apparent absence of SEM bars in some values of panel B indicates that the bar is smaller than the respective symbol. Download Figure S4, PDF file, 0.1 MB. Copyright © 2016 Arias et al. 2016 Arias et al. This content is distributed under the terms of the Creative Commons Attribution 4.0 International license.

10.1128/mSphere.00279-16.5 Figure S5  Replication *in vivo* and in cell culture of MNV NS7 mutants I391V and V330I. (A) MNV titers recovered by reverse genetics following transfection of plasmids containing the cDNA of MNV WT or MNV variants containing different mutations within the NS7 polymerase gene. Statistical significance was determined by a one-way ANOVA test; ns, not significant. Values are the average of the results of at least three independent virus titer determinations performed using virus samples obtained from up to 4 independent cDNA transfections. (B) Replication kinetics of WT and mutant MNVs in RAW264.7 cells infected at an MOI of 5 TCID_50_/cell. Every value in the graph represents the average of results of three independent biological replicates (*n* = 3). Statistical analysis by two-way ANOVA test indicated no significant differences. (C and D) Virus titers (C) and viral RNA levels (D) shed in the feces of five animals inoculated with 3 × 10^2^ TCID_50_ MNV (*n* = 5). Viral samples inoculated in mice were obtained after recovery of genetically defined MNV NS7 variants in BHK-21 cells by reverse genetics followed by 4 serial infections in RAW264.7 cells, as previously described (39). We confirmed by sequencing analysis that the resulting populations harbored only the desired mutations in the NS7 coding gene. Statistical analysis was performed by a two-way ANOVA test (*, *P* < 0.05). In all panels, the dashed line represents the limit of detection in the assay, and the error bars show SEM. The apparent absence of error bars for some values of panel B denotes that the bar was smaller than the corresponding symbol. Download Figure S5, PDF file, 0.05 MB. Copyright © 2016 Arias et al. 2016 Arias et al. This content is distributed under the terms of the Creative Commons Attribution 4.0 International license.

10.1128/mSphere.00279-16.6 Figure S6  Replication kinetics of MNV-3 mutants P72S and E75S during infections in RAW264.7 cells and *in vivo*. (A) RAW264.7 cells were infected at an MOI of 0.01 TCID_50_/cell and viral titers determined at different time points postinfection. Every value is the average of results of three titer determinations, with the error bars representing the SEM for each value. (B) Mice were infected with 10^3^ TCID_50_, and the viral RNA levels shed in the feces were determined at different days postinfection (*n* = 5). In both panels, the dashed line symbolizes the limit of detection, and the statistical significance was determined using a two-way ANOVA test (*, *P* < 0.05; **, *P* < 0.01; ***, *P* < 0.001). Download Figure S6, PDF file, 0.04 MB. Copyright © 2016 Arias et al. 2016 Arias et al. This content is distributed under the terms of the Creative Commons Attribution 4.0 International license.

**FIG 4:**
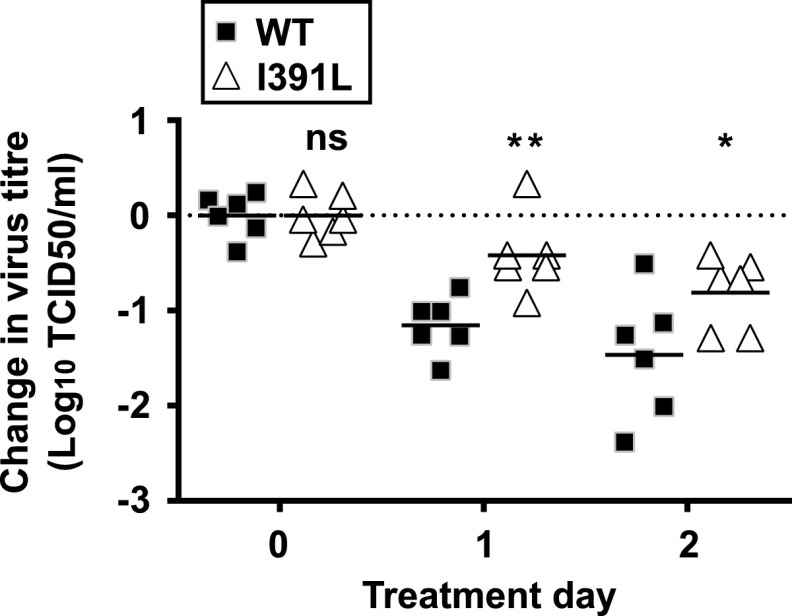
MNV NS7 RNA polymerase mutant I391L is less sensitive to favipiravir treatment *in vivo*. Six animals were infected with 3 × 10^2^ TCID_50_ MNV (*n* = 6). After 7 days, persistently infected mice were subjected to favipiravir treatment twice a day over a period of 4 days. Mouse feces samples were collected, and the titers (TCID_50_ per gram) were determined as previously described (10). The virus titer values represented are relative to the average virus titer at the day 0 time point, collected immediately before the beginning of treatment. Absolute virus titer values at day 0 were 5.80 and 5.51 log_10_ TCID_50_/g stool for the WT and the I391L mutant, respectively. Error bars represent SEM from a two-way ANOVA test (ns, not significant; *, *P* < 0.05; **, *P* < 0.01).

We subsequently investigated whether the reduced replication of I391L in mice could be rescued by a chemical expansion of the quasispecies diversity by favipiravir treatment. We have previously demonstrated that favipiravir elicits antiviral mutagenic activity associated with increased genetic diversity of the viral quasispecies *in vivo* (10). Treatment of mice with favipiravir resulted in decreased virus titers shed by both WT MNV and I391L MNV-infected animals (Fig. 4), suggesting that the dose of favipiravir used elicits antiviral activity against both viruses. Despite this, I391L displayed greater tolerance than the WT to favipiravir during infection *in vivo*, in agreement with observations in cell culture (Fig. 1B).

### The high-fidelity MNV NS7 I391L mutant is transmitted less efficiently than the WT and the S313T mutant *in vivo*.

**FIG 5:**
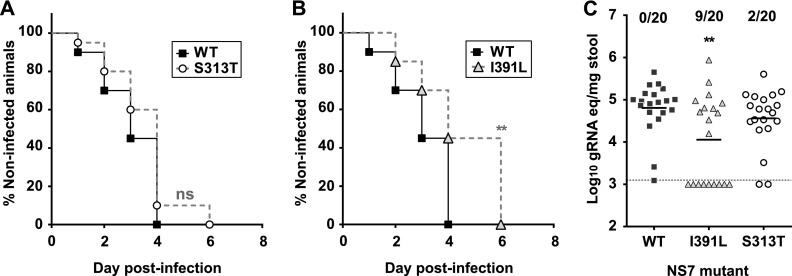
MNV NS7 RNA polymerase mutant I391L shows delayed transmission in susceptible hosts. Virus transmission from infected to uninfected mice was determined as detailed in Materials and Methods. In brief, five naive mice in each cage were housed with one mouse inoculated by oral gavage with 3 × 10^2^ TCID_50_ MNV-3. Every mutant was tested in 4 independent cages (*n* = 20). MNV transmission was determined by qPCR detection of MNV in the feces of naive mice along a series of time points. (A and B) Graphs showing the percentages of noninfected animals along a series of time points (the log rank test was used for statistical analysis). (A) WT and S313T showed no significant (ns) difference in their transmission kinetics between mice. (B) The WT strain was transmitted significantly faster than the I391L mutant (**; *P* < 0.01). S313T also showed significantly increased transmission compared to I391L (not shown in the figure; *P* < 0.05). Animals directly infected by oral-gavage inoculation are not represented. (C) Viral RNA levels found in the feces of noninoculated animals after 4 days in contact with infected mice (*n* = 20). The dashed line represents the limit of detection, and the numbers above the groups represent the numbers of animals shedding viral RNA at levels below the detection limit. Animals directly infected by oral-gavage inoculation are not represented. Data represent results of a two-way ANOVA test (**, 0.001 < *P* < 0.01).

Several studies have found that viral genetic diversity contributes to organ and tissue tropism expansion during viral infections by facilitating avoidance of or escape from different physiological barriers (3, 4, 36). However, there is limited information on whether genetic diversity contributes to virus transmission between susceptible hosts. Recent studies suggest that reduced polymerase replication fidelity may be a significant determinant linked to the pandemic expansion of recent HuNoV GII.4 isolates (21) and that person-to-person transmission is more efficient for GII.4 pandemic strains than for nonpandemic isolates (19, 20). To determine whether the high-fidelity I391L NS7 mutant had altered transmission *in vivo*, we established an MNV transmission model whereby one mouse per cage was orally inoculated and transmission to five sentinel littermates was monitored by quantification of viral RNA in the feces. Since a defect in transmission could be attributed to delayed replication in I391L-infected mice, we also included the S313T mutant as it exhibits WT-like polymerase fidelity (Fig. 1D) but slower replication *in vivo*, to an extent similar to that seen with the I391L mutant (Fig. 3A and B). We found that transmission rates for the I391L mutant were significantly lower than those found for the WT and S313T (Fig. 5), suggesting that genetic diversity is linked to efficient virus transmission between hosts. Reduced transmission in the I391L-infected group was associated with lower viral RNA levels shed in the feces of sentinel animals (see Fig. S7 in the supplemental material). Hence, changes in polymerase fidelity may impact the rate of transmission of noroviruses within a susceptible population of naive hosts, providing additional support to recent studies indicating that the pandemic HuNoV GII.4 isolates possess polymerases with lower fidelity (21, 28).

10.1128/mSphere.00279-16.7 Figure S7  Viral RNA levels shed in the feces of sentinel mice in contact with orally inoculated mice. Naive mice in contact with WT-infected mice shed greater levels of viral RNA than mice in contact with S313T or I391L mice over the duration of the experiment (*n* = 20). The dashed line indicates the detection limit for this assay. Data represent results of a two-way ANOVA test (*, *P* < 0.05; **, *P* < 0.01). Download Figure S7, PDF file, 0.03 MB. Copyright © 2016 Arias et al. 2016 Arias et al. This content is distributed under the terms of the Creative Commons Attribution 4.0 International license.

In this study, we demonstrated that a high-fidelity norovirus mutant has reduced replication *in vivo* accompanied by reduced rates of transmission between hosts. This report builds on our previous investigations demonstrating that norovirus replication fidelity and intrahost genetic diversity influence viral pathogenesis (10). Together, these results are highly significant in light of understanding the influence of replication fidelity on factors that affect the emergence of new norovirus variants with pandemic potential. Given the recent development of potential cell culture propagation methods for HuNoVs (37, 38), the identification of fidelity determinants in NS7 could also contribute to the design of genetically stable attenuated vaccines, as has been proposed for other viruses (8).

## MATERIALS AND METHODS

### Ethics.

Studies with mice were performed in the Department of Pathology Biostatistics Unit (BSU) (PCD 80/2802) after ethical review by the University of Cambridge Review Panel and subsequent approval by the United Kingdom Home Office (PPL70/7689). All animal procedures and care conformed strictly to the United Kingdom Home Office Guidelines under the Animals (Scientific Procedures) Act 1986.

### Animal infections and antiviral treatment.

Male C57BL/6 mice (4 to 5 weeks old) were orally infected with known amounts of MNV-3 as previously described (10) and as stated in the text. Fecal samples were collected at different time points, and the presence of infectious particles and viral RNA was determined as described in reference 10. For the determination of viral RNA levels in tissues, animals were euthanized at 48 h postinfection and the cecum, colon, and mesenteric lymph node harvested.

Mice infected with MNV-3 were treated with favipiravir following protocols previously described in reference 10. Briefly, 1 week after inoculation, persistently infected animals were treated twice a day by oral gavage with favipiravir (600 mg/kg of animal body weight/day) for 4 days. Fecal samples were collected at different times during the course of treatment and virus titers and RNA levels quantified.

### Viral transmission experiments.

To determine MNV transmission *in vivo*, we scored the proportion of infected mice within the population of noninoculated animals in contact with mice infected by oral-gavage inoculation. One mouse was inoculated per cage and was housed with five contact uninfected sentinel mice. We determined the presence of MNV RNA in the feces of naive mice at different time points postinfection (days 1, 2, 3, 4, and 6).

### Cells, infections, and reverse genetics recovery of viruses.

Procedures for the cultivation of cells and MNV infections have been previously described (10). RAW264.7 murine leukemia macrophage cells were used for the propagation and titration (TCID_50_ assay) of the MNV-1 and MNV-3 mutants used in this study. All the different cell lines were cultured in Dulbecco’s modified Eagle medium (DMEM) with 10% FCS, 100 U/ml penicillin, and 100 mg/ml streptomycin (complete DMEM) and were maintained at 37°C with 5% CO_2_.

The wild-type strain and NS7 mutants MNV-1 and MNV-3 used in this study were obtained after reverse genetics recovery of infectious virus as previously described (10, 27). Briefly, BHK-21 cells were transfected with plasmids containing a T7 promoter followed by full-length genomic MNV cDNA encoding different NS7 mutants. Then, transfected cells were infected with helper fowlpox virus expressing recombinant T7 polymerase (FPV-T7) as previously described in reference 27. The resulting population was titrated and used as a passage 0 stock. All the experiments carried out in this study involved NS7 mutants in the context of an MNV-3 genome, with the exception of a limited number of NS7 mutant virus recoveries described in Table 1 that were generated in the context of an MNV-1 genome.

### Cell culture infections in the presence of mutagenic compounds.

Experiments performed to determine levels of sensitivity to different mutagenic compounds were carried out using RAW264.7 cells. A total of 4 × 10^5^ cells per well were seeded on 24-well plates, incubated for 3 h at 37°C and 5% CO_2_ to allow attachment to the plate, and then infected with different MNV mutants at an MOI of 0.01 TCID_50_/cell. Cells were incubated at 37°C and 5% CO_2_ for 1 h; the supernatants were then removed and cells washed once with complete DMEM, before addition of 1 ml of complete DMEM containing mutagenic compounds at the indicated concentrations. Infections were harvested at 42 h and virus progeny released through 2 consecutive cycles of freeze-thawing.

### Viral RNA extraction, reverse transcription-PCR (RT-PCR) amplification, quantitative PCR (qPCR), and mutation frequency analysis of virus populations.

Viral RNA was extracted from 100 µl of viral samples (either supernatant from lysed infected cultures or phosphate-buffered-saline [PBS]-resuspended feces from animals) using a GenElute RNA miniprep purification system following protocols provided by the manufacturer. Viral RNA was quantified using a two-step qPCR approach following protocols described previously (27).

For the calculation of mutation frequency in any virus population, we followed protocols previously described (10). We analyzed the mutation frequency of mutant and WT populations obtained after eight passages in RAW264.7 cells to allow sufficient rounds of replication and accumulation of mutations to facilitate the quantitative analysis. Briefly, 4 µl of purified RNA was subjected to reverse transcription in a 20-µl final volume using SuperScript III (Roche) as indicated by the manufacturer. cDNA (3 µl) was then PCR amplified using high-fidelity Hot Start KOD DNA polymerase (Toyobo) and primers spanning MNV-3 genomic positions 951 to 3395. Positive PCR band products were excised from an agarose gel and purified with EconoSpin columns (Epoch) and then directly ligated to plasmid PCR Blunt using a Zero Blunt PCR cloning kit (Life Technologies). Positive *Escherichia coli* colonies were identified by PCR screening using primers flanking the vector-cloning site and GoTaq polymerase (Promega). The resultant PCR products corresponding to individual MNV cDNA clones were sequenced, and the mutation frequency in each population was calculated.

### Statistical analysis.

Statistical significance was examined using GraphPad Prism as described in the figure legends. For statistical analysis of mutation frequencies, a Mann-Whitney test was used that compares the ranked scores of the numbers of mutations found in individual clones grouped by population (Mann-Whitney test; 0.01 < *P* < 0.05, **), as previously described (5).
