# Supplementary figures and images for: Norovirus Polymerase Fidelity Contributes to Viral Transmission In Vivo
Source: mSphere. 2016 Oct 19;1(5):e00279-16. doi: 10.1128/mSphere.00279-16 (PMC5071534; doi:10.1128/mSphere.00279-16)

Figure S1

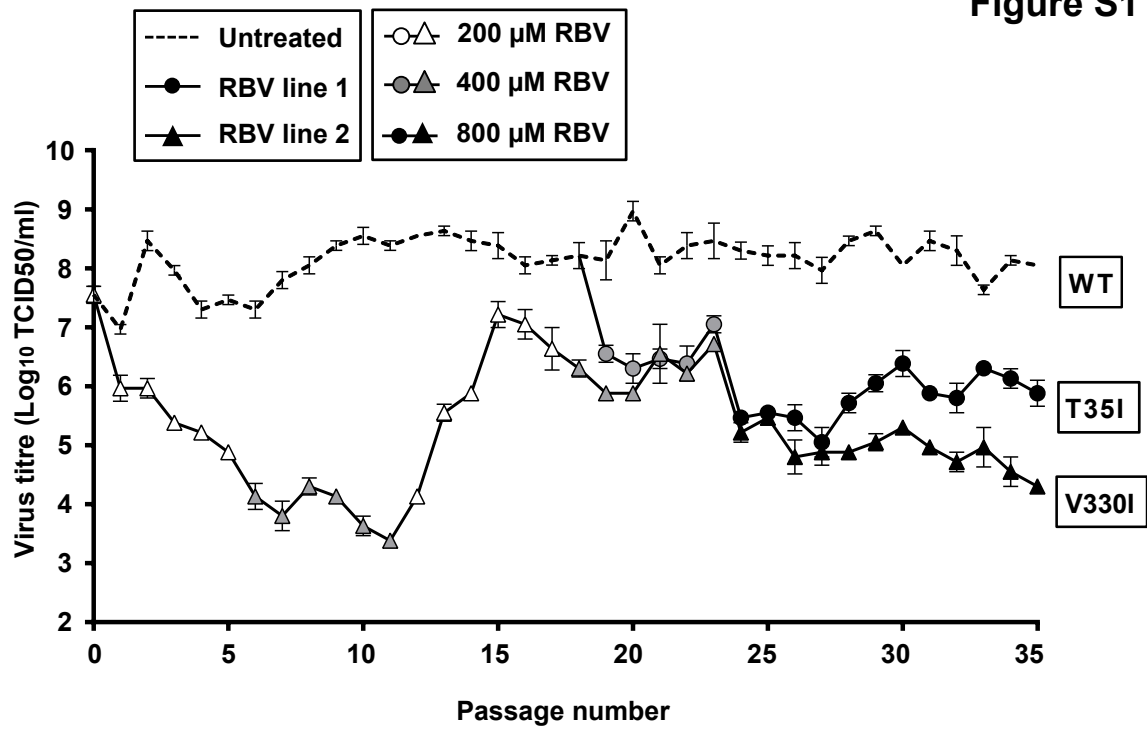

Supplement: Figure S1 [file sph005162170sf1.pdf]

Figure S2

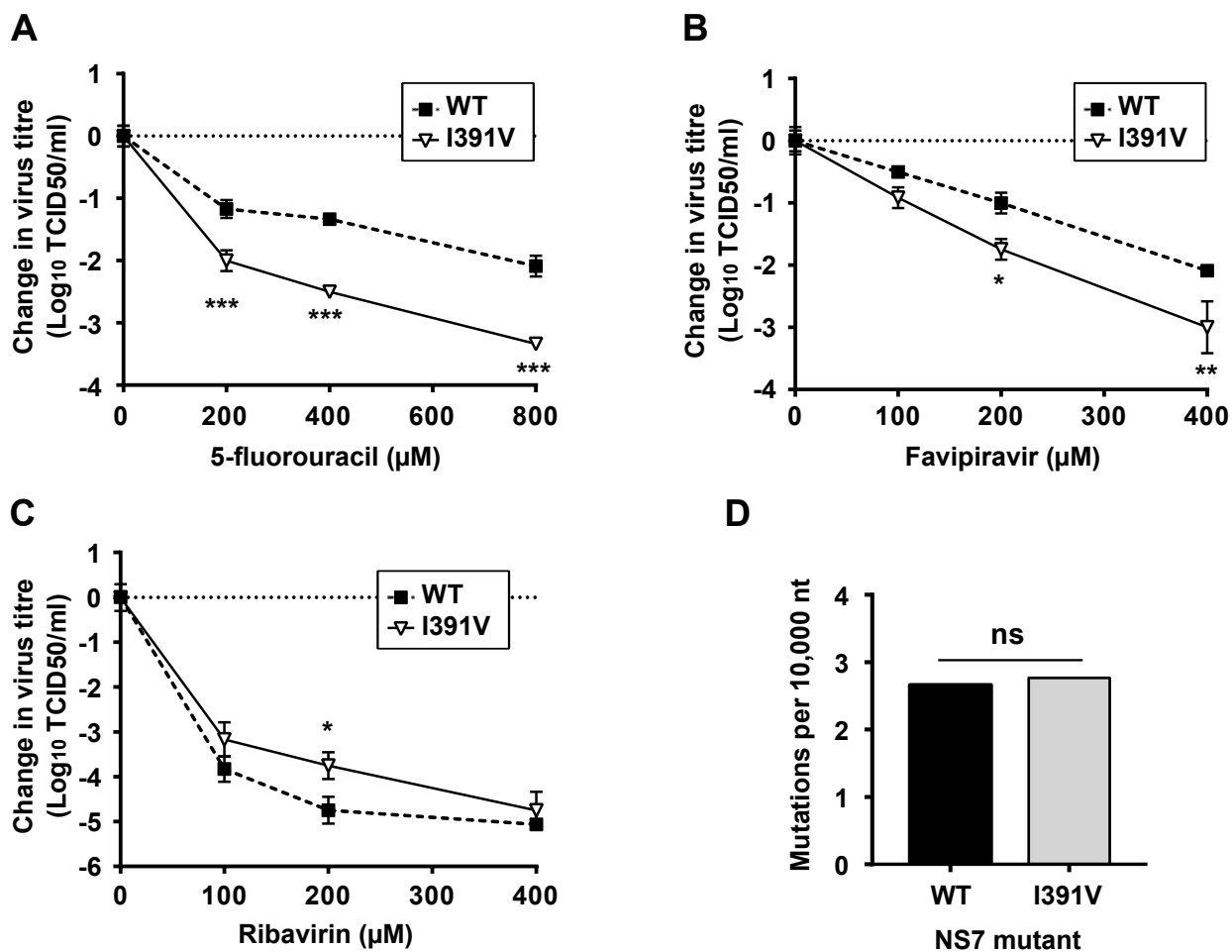

Supplement: Figure S2 [file sph005162170sf2.pdf]

Figure S3

A

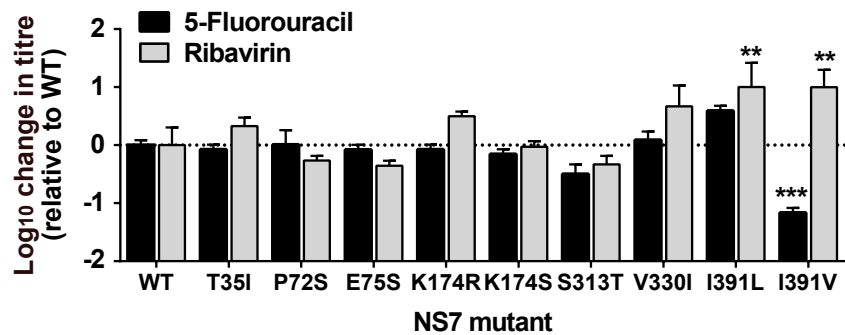

B

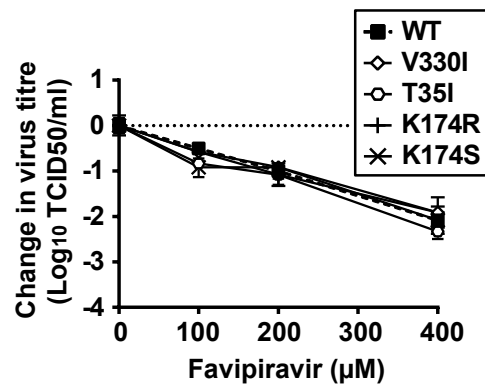

Supplement: Figure S3 [file sph005162170sf3.pdf]

Figure S4

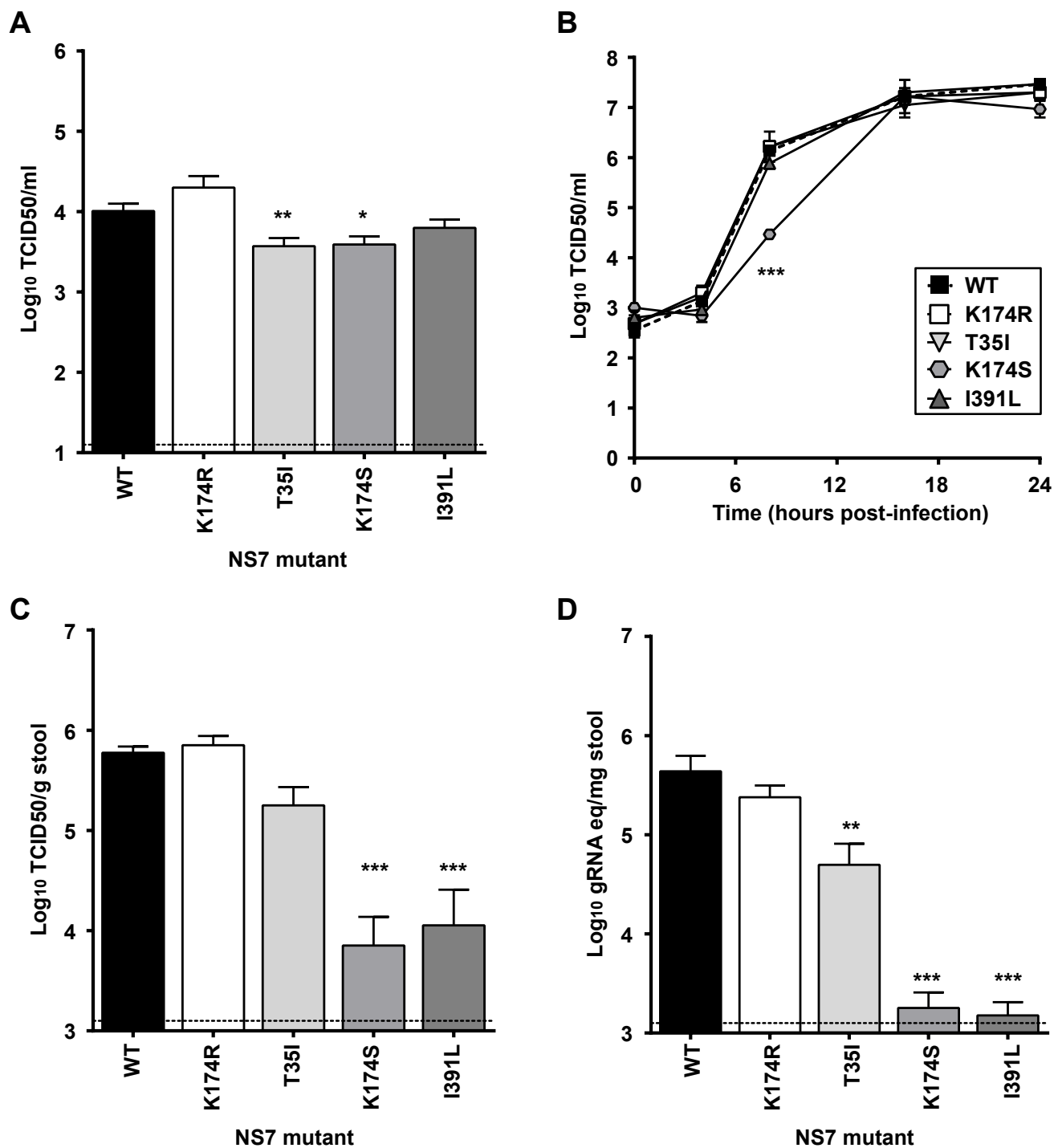

Supplement: Figure S4 [file sph005162170sf4.pdf]

Figure S5

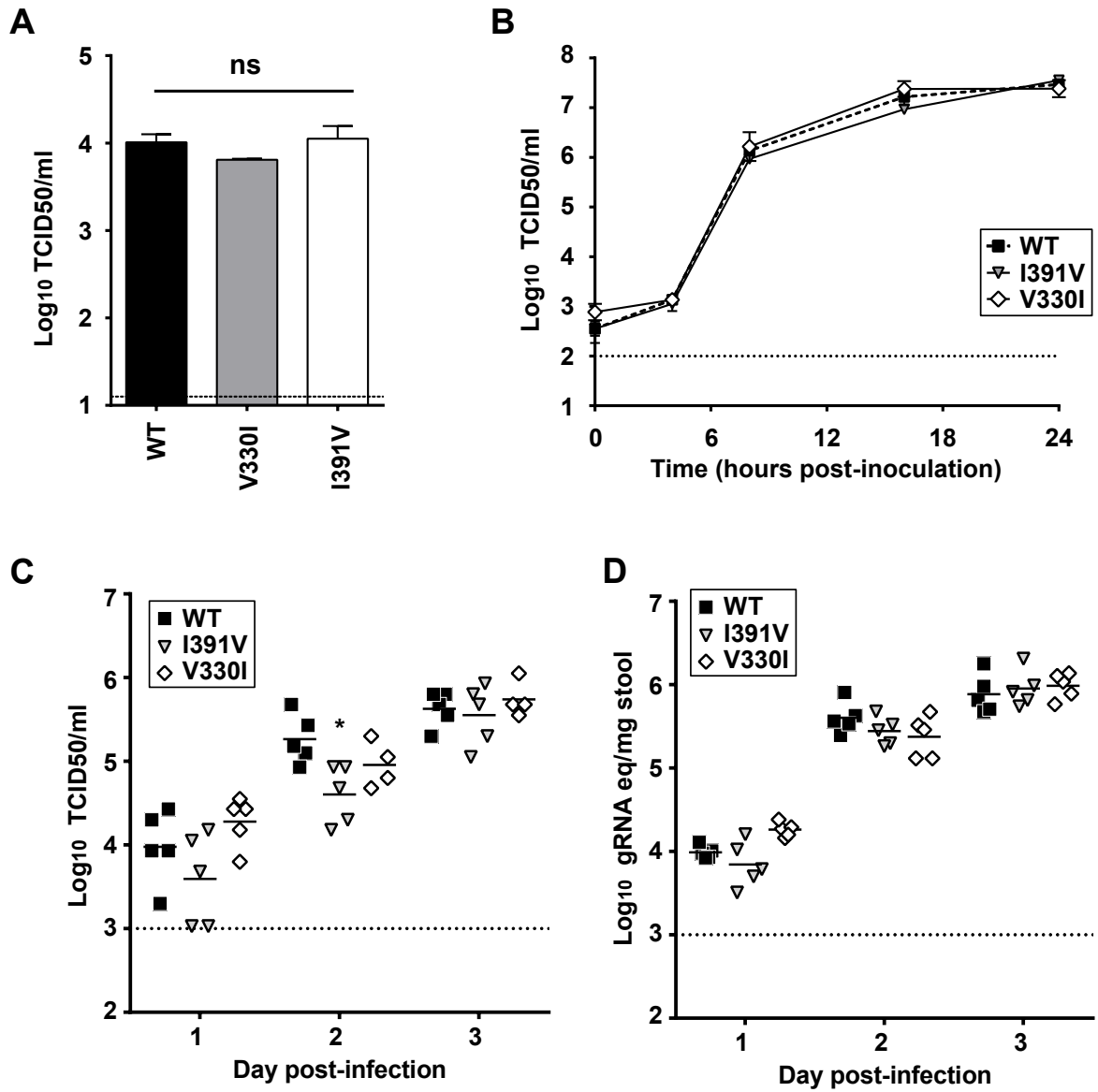

Supplement: Figure S5 [file sph005162170sf5.pdf]

Figure S6

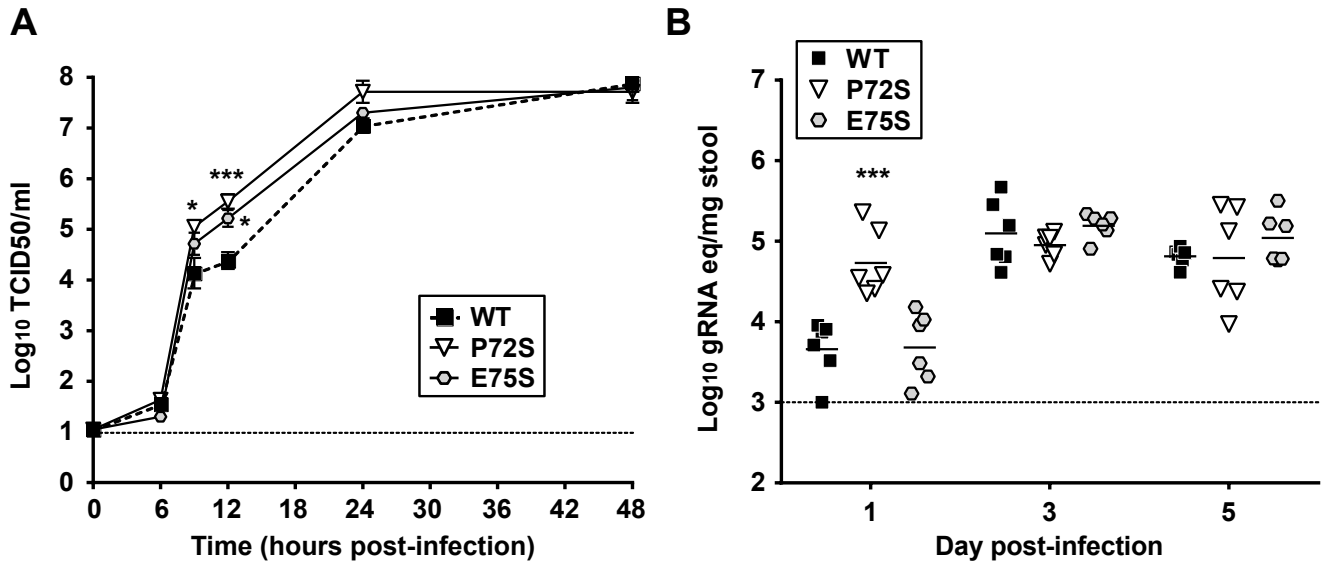

Supplement: Figure S6 [file sph005162170sf6.pdf]

Figure S7

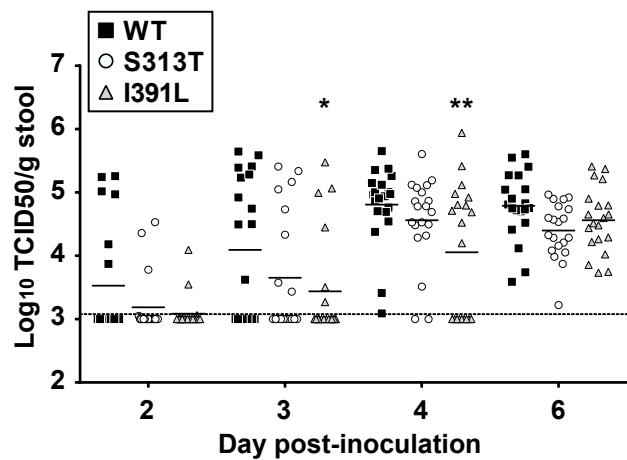

Supplement: Figure S7 [file sph005162170sf7.pdf]
