# Supplementary material for: Norovirus Polymerase Fidelity Contributes to Viral Transmission In Vivo
Source: mSphere. 2016 Oct 19;1(5):e00279-16. doi: 10.1128/mSphere.00279-16 (PMC5071534; doi:10.1128/mSphere.00279-16)
Supplement: Table S1 [file sph005162170st8.docx]

**Table S1. Mutation frequency analysis of NS7 mutant virus populations**

| Mutant virus passage 8 | Number of mutations^a^ | Total nucleotides | Number of clones analysed^b^ | Ts^c^ | Tv^d^ | Indels^e^ | Mutation frequency^f^ | Relative  fidelity^g^ |
| --- | --- | --- | --- | --- | --- | --- | --- | --- |
| WT | 34 | 127,870 | 60 | 26 | 7 | 1 | 2.7 x 10^-4^ | 1 |
| S313T | 23 | 71,844 | 34 | 20 | 2 | 1 | 3.2 x 10^-4^ | 0.8 |
| I391L | 10 | 117,540 | 54 | 10 | --- | --- | 0.9 x 10^-4^ | 3.0 |
| I391V | 20 | 72,242 | 34 | 19 | 1 | --- | 2.8 x 10^-4^ | 0.9 |

Mutation frequencies are calculated by analysing individual clones sequences isolated from MNV-3 populations after molecular cloning of PCR amplification products. Different NS7 polymerase mutants were analysed: wild type (WT), I391L, I391V and S313T. Mutation frequencies in I391L populations were 3-fold lower than in WT populations.

^a^ Number of mutations found during the analysis of individual clones isolated from NS7 mutant viral quasispecies after 8 passages in cell culture. Repeated mutations are only considered once for the analysis as previously described in Gnadig et al PNAS 2012; 109:E2294.

^b^ Total number of nucleotides analysed for each mutant virus population

^c^ Transitions

^d^ Transversitions

^e^ Insertions and deletions

^f^ Mutation frequency. This number is calculated from dividing the total number of mutations found in individual clones by the total number of nucleotides analysed

^f^ Relative fidelity in a given NS7 mutant is expressed as the ratio between the mutation frequency found in WT population (2.7 x 10-4 mutations/nucleotide analysed) and the mutation frequency in the mutant population
